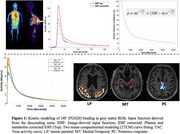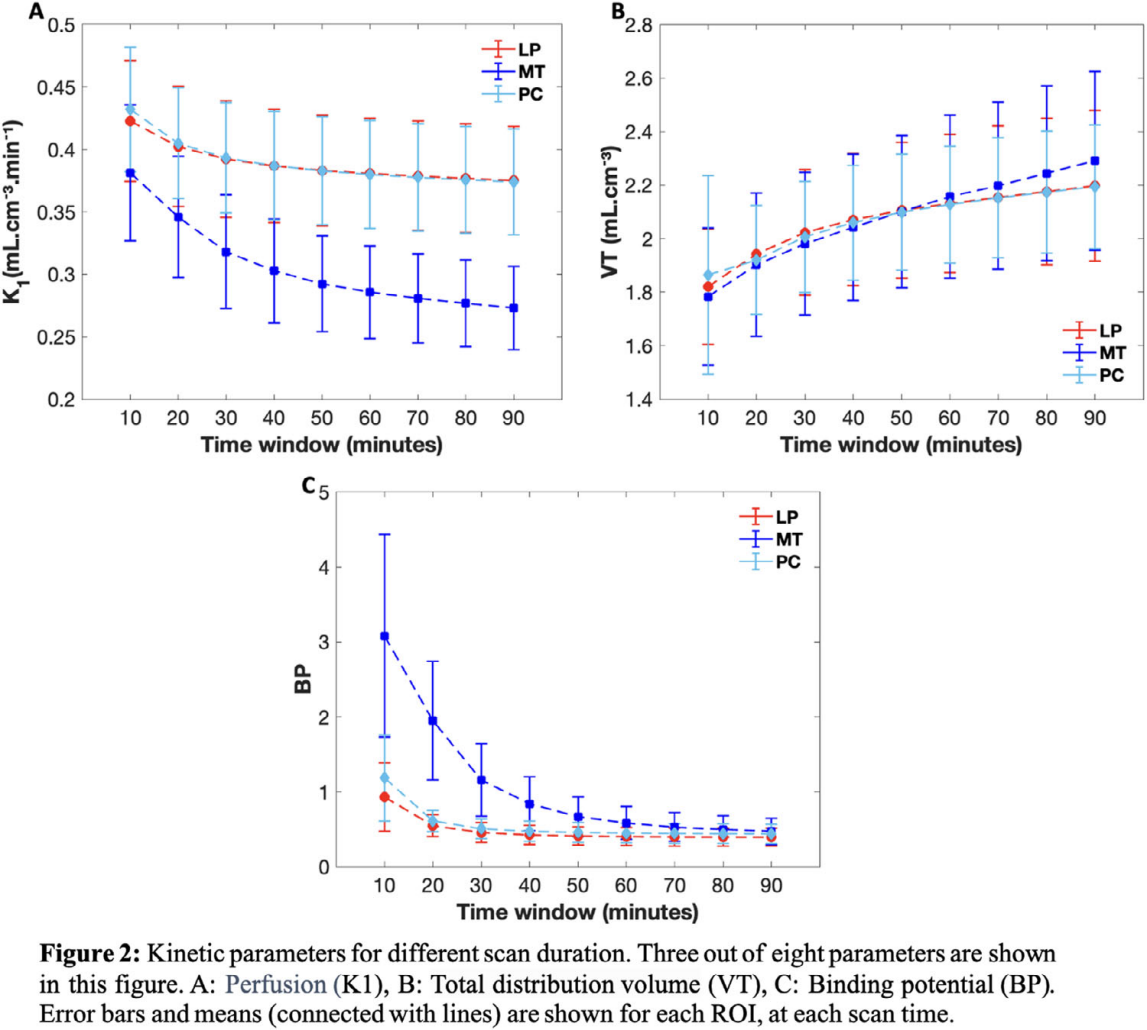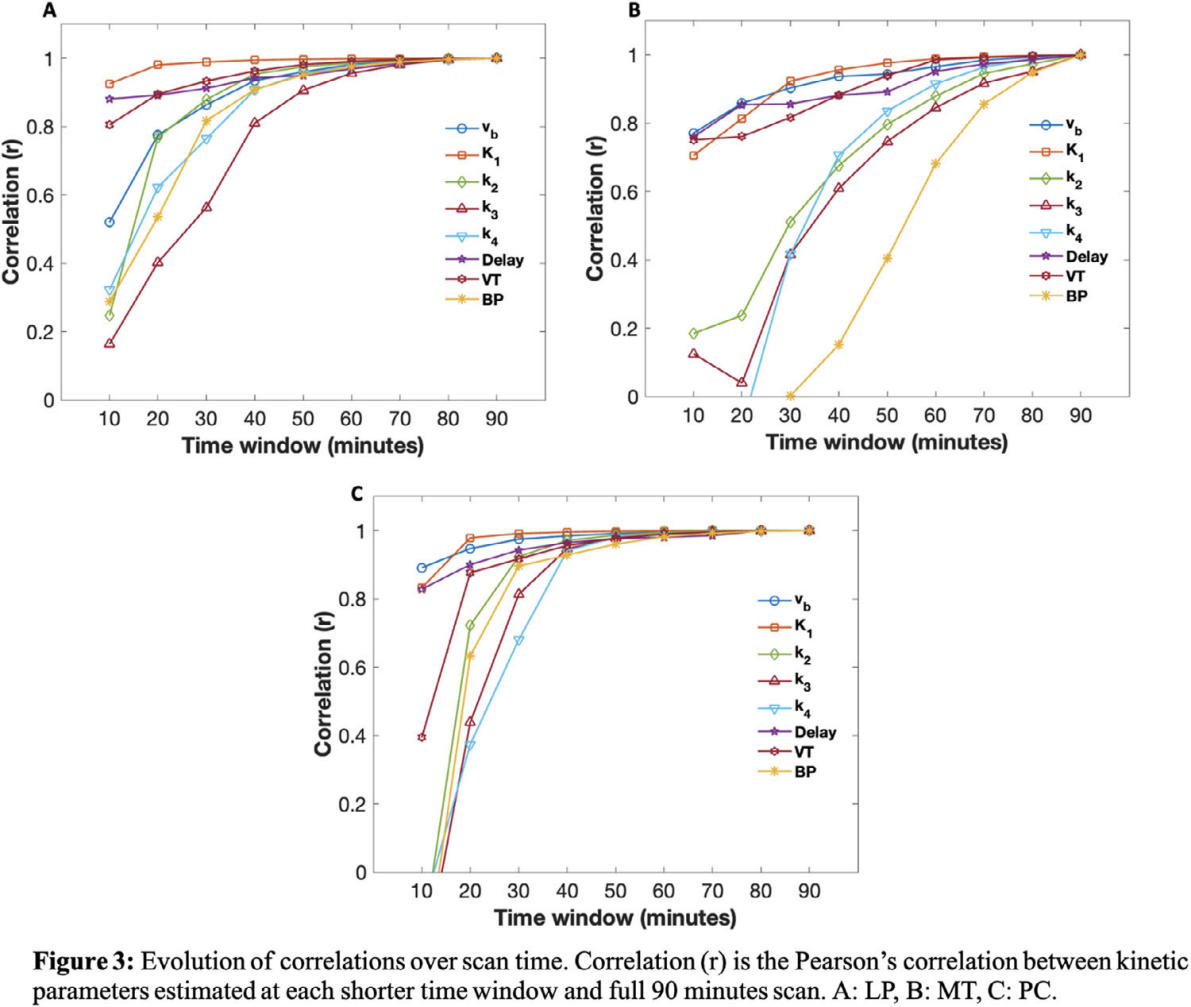# Non‐invasive quantification of [^18^F]‐PI‐2620 binding to tau deposits in the brain using an image‐derived input function

**DOI:** 10.1002/alz.088652

**Published:** 2025-01-09

**Authors:** Anjan Bhattarai, Emily Nicole Holy, Yiran Wang, Benjamin A. Spencer, Guobao Wang, Charles S. DeCarli, Audrey P. Fan

**Affiliations:** ^1^ University of California Davis, Davis, CA USA

## Abstract

**Background:**

This study quantified tau binding in the brain with ^18^F‐PI2620 PET using a non‐invasive Image‐Derived Input function(IDIF), derived using a new total‐body EXPLORER PET/CT scanner (Spencer et al.,2021). Additionally, we explored how PET scan duration influences the quantification of kinetic parameters across brain regions of interest(ROIs) that are vulnerable in Alzheimer’s Disease.

**Method:**

The study cohort included 15 individuals (10 cognitively unimpaired, 3 mild cognitive impairment, and 2 AD; ages=68‐92 years; all tau negative) from the UC Davis ADRC. Dynamic total‐body ^18^F‐PI‐2620 PET images were acquired over 90 minutes using the uEXPLORER (resolution=2.344 mm isotropic voxels, framing protocol:30×2s,12×10s,7×60s,16×300s). Brain cropped PET images were motion corrected (Jenkinson et al.,2002), and linearly registered to individual T1‐weighted(T1W) MRIs (Jenkinson & Smith, 2001). Grey matter ROIs, including the medial temporal(MT), posterior cingulate(PC), and lateral parietal(LP) cortices, were obtained parcellating T1W images (Desikan et al.,2006).

Dynamic time activity curves for each brain ROI were fitted to a reversible two‐tissue compartmental model(2TCM), accounting for delay, using a subject‐specific IDIF(plasma and metabolite‐corrected) derived from the descending aorta of the same images (Figure 1). ROI‐specific micro and macro kinetic parameters were estimated. Logan graphical analysis (Logan,2000) was also used to estimate total distribution volume(V_T_).

**Result:**

2TCM with IDIF demonstrated high‐quality fits of ^18^F‐PI2620 binding across grey matter ROIs (Figure 1). Perfusion (K_1_) was observed to be reduced in MT compared to PC and LP (Figure 2A).

All kinetic parameters remained relatively stable after the 60‐minute scan window across all ROIs. Notably, K_1_ showed stability after 30 minutes of scan duration across all ROIs (Figure 2 and 3).

A strong correlation was observed between VT estimated using 2TCM and Logan plot analysis, across all ROIs LP(r=1.00, p<0.001), MT(r=0.96, p<0.001), PC(r =1.00, p<0.001).

Additionally, significant negative correlation was observed between binding potential and perfusion across all ROIs (r=‐0.40, p=0.007).

**Conclusion:**

This study highlights the utility of a non‐invasive descending aorta IDIF derived using a total‐body EXPLORER PET/CT scanner to quantify ^18^F‐PI‐2620 grey matter kinetics. Preliminary findings suggest that a 60‐minute scan window may be sufficient for reliable quantification of kinetic parameters, while a 30‐minute scan time may suffice for perfusion alone. These findings warrant validation in a cohort involving tau positive individuals.